# The Changes of Cheek Line (Lateral) and Face Line (Frontal) after Correction of Mandibular Prognathism

**DOI:** 10.1155/2018/4926528

**Published:** 2018-06-11

**Authors:** Yu-Chuan Tseng, Jung-Hsuan Cheng, Michael Yuan-Chien Chen, Kwei-Jing Chen, Chun-Ming Chen

**Affiliations:** ^1^Department of Orthodontics, Kaohsiung Medical University Hospital, Kaohsiung, Taiwan; ^2^School of Dentistry, College of Oral Medicine, Kaohsiung Medical University, Kaohsiung, Taiwan; ^3^School of Dentistry, College of Medicine, China Medical University, Taichung, Taiwan; ^4^Department of Dentistry, China Medical University Hospital, China Medical University, Taichung, Taiwan; ^5^Department of Oral and Maxillofacial Surgery, Kaohsiung Medical University Hospital, Kaohsiung Medical University, Kaohsiung, Taiwan

## Abstract

**Objective:**

The purpose of the present study was to investigate the improvements of facial profile and postoperative stability by single mandibular setback surgery.

**Materials and Methods:**

The study included twenty-seven patients who underwent mandibular prognathism correction by sagittal split ramus osteotomy (SSRO). Cephalometric radiograms (lateral and frontal) were collected and analyzed at three intervals: preoperatively (T1), immediately postoperatively (T2), and final follow-up postoperatively (T3). The lateral and frontal cephalometric parameters were measured. The immediate postoperative change (T21), postoperative stability (T32), and final surgical change (T31) were calculated and analyzed. The null hypothesis is that postoperative stability (T32) was not significantly correlated to amount of mandibular setback (T21).

**Results:**

The immediate postoperative change (T21) of menton (Me) was significantly backward 8.7 mm. In the final postoperative change (T31), average chin points anterior movements were approximately 0.32 mm. Investigating frontal appearance, inter ramus posterior (InterRp) and intergonion (InterGo) widths were significantly increased with 1.8 and 2.2 mm, respectively. Bilateral ramus angles were not significantly increased, about 1°. The horizontal Me (T32) had significant correlation (*p* = 0.028) with amount of setback (T21). Therefore, null hypothesis is rejected.

**Conclusion:**

Postoperative relapse was significantly correlated to the amount of setback. The frontal transverse changes (InterRp and InterGo) were significantly increased.

## 1. Introduction

Not all unfavorable dentition, occlusion, and morphology are caused by simple malalignment of teeth. Potential abnormal skeletal development problems are also a cause of abnormal occlusion. For instance, Angle's Class III malocclusion is relatively common among Asians (15%) [1, 2] than among Caucasians (<5%) [3, 4]. Mandibular prognathism is usually accompanied with a severe Angle's Class III malocclusion. Orthognathic surgery for the treatment of mandibular prognathism has been improved in many different ways from the extraoral approach to the intraoral approach. Regardless of the kind of surgery performed to treat mandibular prognathism, osteotomy position of the mandible could affect the postoperative stability between the proximal and distal segments. Sagittal split ramus osteotomy (SSRO) is one of the common methods currently used for mandibular setback operation. In SSRO technique, miniplates or miniscrews are used to fix the proximal and distal segments and instead of intermaxillary fixation.

Numerous studies [5–8] have reported the profile's changes after SSRO in the treatment of mandibular prognathism. Cheek line [9] is a curved and charming line, which is located between the nose and the cheek bone. Clinical observations have revealed that the cheek lines were advanced slightly when their mandibles were set back. The pterygomasseteric sling (PMS), the powerful elevators of the jaw, formed by the medial pterygoid and masseter muscles. Therefore, the PMS area changes of the ramus maybe influence the postoperative stability. Moreover, patients are also concerned with their postoperative frontal appearance, not only the lateral profile. The purpose of this study is to investigate (1) the postoperative mandibular stability, (2) the changes of cheek line, and (3) PMS frontal appearance.

## 2. Materials and Methods

Twenty-seven patients (17 males and 10 females; mean age, 24.07 ± 3.46 years) who needed surgical correction of mandibular prognathism were treated at the Oral and Maxillofacial Surgery Department of the China Medical University Hospital. The surgery was carried out using only SSRO setback. The inclusion criteria for participants in the study were as follows: (1) All the patients who participated in the study must have mandibular prognathism. (2) There should be no craniofacial anomalies in all patients. (3) Patients with a history of trauma or recognized syndromes were excluded.* (4) There were some patients with slight facial asymmetry without the need of genioplasty*. Three cephalometric radiographs were obtained 1 month preoperatively (T1), immediately (2 days) after surgery (T2), and over 6-month final postoperative follow-up (T3). The immediate postoperative change (T21), postoperative stability (T32), and final surgical change (T31) were calculated and analyzed. The following points were identified: sella (S), nasion (N), menton (Me), pognion (Pog), pognion at the soft tissue (PogS), pronasale (Prn: tip of nose), porion (Po), orbitale (Or), antegonial notch (Ag),* gonion (Go)*, and sigmoid notch (Sm).

**Figure 1:**
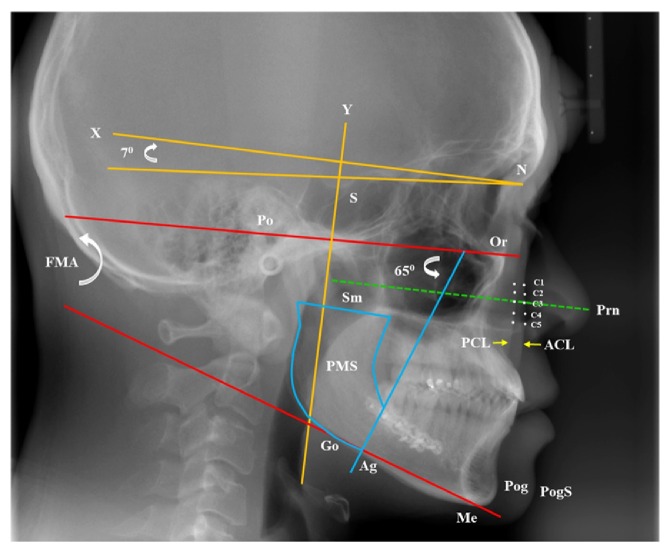
Cephalometric landmarks; linear and angular measurements. N: nasion, S: sella, Prn: pronasale, Me: menton, Pog: pognion, PogS: soft tissue of Pog, Po: porion, Or: orbitale, Sm: sigmoid notch, Ag: antegonial notch. *x*-axis (horizontal line: 7° to NS line), *y*-axis (vertical line through S). FH plane: a line connecting Po to Or. Pterygomasseteric sling (PMS) plane: a line through Ag point 65° to FH plane. Cheek line (yellow arrow line): anterior cheek lines (ACL) and posterior cheek line (PCL). Cheek point (C1–C5): C3 through Prn vertical line* (green-dashed line)* to *y*-axis. C1: +6 mm, C2: +3 mm, C3: 0, C4: −3 mm, and C5: −6 mm.* Frankfort-mandibular plane angle (FMA) is the angle between Frankfort horizontal plane and gonion-menton (Go-Me) plane.* PMS areas (blue color): ramus area between PMS line and a line through Sm parallel to FH plane.

For analysis, the *x*-*y* coordinate axis (Figure 1) was constructed. The horizontal axis (*x*-axis) [10] had its origin at point N at an angle of 7 degrees (upward) with the NS line. The vertical line was perpendicular to it through S as the vertical axis (*y*-axis). Frankfurt horizontal (FH) plane was a line connecting Po to Or.* In Figure 1, a baseline through pronasale (Prn: tip of nose) was vertical to y-axis and intersected with anterior cheek line (ACL) and posterior cheek lines (PCL; origin point: C3).* The cheek points were marked for every 3 mm above or below the C3 point (C1: +6 mm, C2: +3 mm, C4: −3 mm, and C5: −6 mm). Similar to Lee and Yu's report [11], a pterygomandibular sling (PMS) plane was proposed at Ag point at 65° angle relative to FH plane. PMS areas were between PMS line and a line through Sm parallel to FH plane (Figure 1).* Frankfort-mandibular plane angle (FMA) is the angle between Frankfort horizontal plane and gonion-menton (Go-Me) plane. Thirteen patients (nonhigh FMA) in whom the FMA measured ≦29*°* and 14 patients in whom the FMA measured >29*°* were included in the high FMA group [12]*.

**Figure 2:**
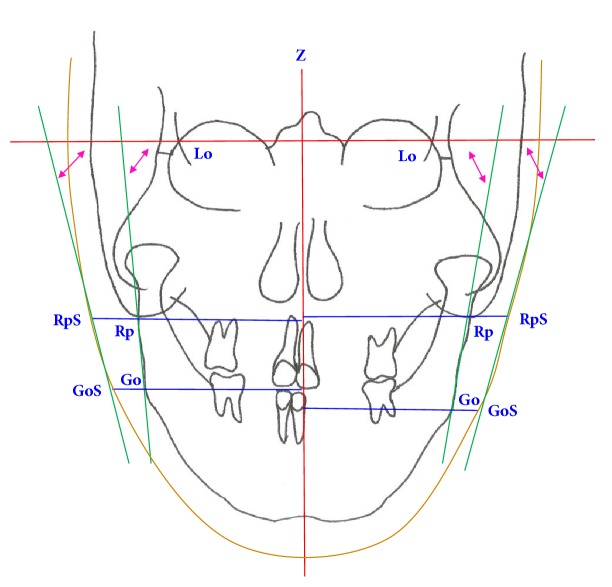
Lo: lateral orbital, Rp: ramus posterior, RpS: ramus posterior at the soft tissue, Go: gonion, GoS: gonion at the soft tissue *z*-axis. Red line: midsagittal line perpendicular to the horizontal plane (Lo-Lo). Green line: skeletal (Rp-Go) and soft tissue (RpS-GoS) ramus lines. Ramus angle (hard tissue): angle between Lo-Lon line and Rp-Go line. Ramus angle (soft tissue): angle between Lo-Lon line and RpS-GoS line.

In the posteroanterior film, the horizontal plane was connected to the bilateral Lo point. The *z*-axis was a midsagittal line perpendicular to the horizontal plane (Figure 2). The landmarks were the lateral orbital (Lo), ramus posterior (Rp), soft tissue of ramus posterior (RpS), gonion (Go), and soft tissue of gonion (GoS). Lo is the intersection of the lateral orbital contour with the innominate line. Rp is the most lateral inferior point, where the outline of the mastoid process crosses the neck of the condyle. Go is the most lateral inferior point of the proximal segment. The angular and linear measures (InterRp and InterGo distances) of the landmarks corresponded with those of the coordinate system. The skeletal (soft tissue) ramus angle is the angle between the Rp-Go (RpS-GoS) line and the horizontal plane. The correlations of soft-to-hard tissue movement were measured.

Relapse is defined as the forward movements of Me. Paired *t*-test was used in detecting mean changes in the variables between the different stages. Pearson's correlation was calculated between the surgical change and related variables, with *p* < 0.05 for the significant test. The null hypothesis is that postoperative stability (T32) was not significantly correlated to amount of mandibular setback (Me). This retrospective study was approved by the human investigation review committee at the China Medical University Hospital (CMUH105-REC2-146).

## 3. Results

The immediate postoperative changes (T21) of Me was significantly backward 8.7 mm and downward 0.4 mm without significance (Table 1). The immediate postoperative change (T21) of Pog was significantly backward 8.1 mm and upward 0.4 mm without significance. PogS was significantly backward 7.2 mm and downward 1.0 mm without significance. Investigating postoperative stability (T32), Me was significantly forward 3.7 mm and upward 1.0 mm without significance. In the final surgical change (T31), Me was statistically significantly backward 5.0 mm and upward 0.7 mm without significance. The reduction of PMS's area (Table 2) was 55.5 mm^2^ (3.6%). Pog was statistically significantly backward 5.2 mm and upward 1.4 mm. PogS was statistically significantly backward 5.5 mm and upward 1.1 mm without significance.

In the final surgical change (T31), cheek points ranged from 0.21 to 0.36 mm (mean = 0.32 mm) advancement without significance (Table 2).* There is no significant change (T31) in the total FMA, high FMA, and nonhigh FMA groups. In the nonhigh FMA group, Me (T32) was forward 3.26 mm. In the high FMA group, Me (T32) was forward 4.05 mm. There is also no significant difference in the skeletal relapse between nonhigh FMA and high FMA patients.*

Investigating final changes of frontal landmarks (T31), only left sides of horizontal Rp and vertical Go were significantly increased with 1.02 mm and decreased with 2.43 mm, respectively (Table 3). InterRp and InterGo distances were significantly increased with 1.8 mm and 2.2 mm (Table 4). However, InterRpS and InterGoS widths were not significantly increased with 1.0 and 1.9 mm, respectively. Bilateral ramus angles were increased about 1°. Bilateral soft tissues of ramus angles were not significantly increased, 1.9° (right side) and 1.1° (left side).

Pearson's test (Table 5) showed that horizontal Me (T32) had significant correlation (*p* = 0.028) with amount of setback (T21). Therefore, null hypothesis is rejected. Postoperative relapse was significantly correlated to the amount of setback. The vertical Me (T32) had significant correlation with horizontal change of left Go (T31) and vertical changes of right Rp (T31), right Go (T31), left Rp (T31), InterGo distance (T31), and left ramus angle (T31).* FMA (T31) had no significant correlation with Me (T32).* The soft-to-hard tissue ratios of the final surgical change (T31) were significantly correlated as follows: PogS/Pog (1.05 : 1; *p* < 0.001). The average cheek points/Pog (0.06 : 1), InterRpS/Pog (0.19 : 1), and InterGoS/Pog (0.36 : 1) were not significantly correlated.

## 4. Discussion

Movements of the proximal and distal segments after mandibular ramus osteotomies are noted to affect the postsurgical skeletal stability. Postsurgical skeletal stability is a crucial factor that affects not only the difficulty of orthodontic treatment but also the facial improvements of the patient. In cases of postsurgical skeletal instability, postoperative duration of orthodontic treatment will increase and improvements in the patient's profile can be mitigated.

Komori et al. [13] suggested using skeletal fixation, which involves the use of circummandibular wiring and maxillary interdental alveolar bone between the incisor and canine, to perform suspension wiring fixation and resist displacement. However, the suspension wiring fixation technique is intramaxillary and the suspension wiring can only be removed through surgery under anesthesia after fixation for a certain period of time, which is an inconvenience to the patients. Currently, SSRO mostly used the rigid fixation, which comprises bicortical screw or miniplate fixation techniques to stabilize the proximal and distal segments of the mandible. Therefore, intra- or intermaxillary fixation is not necessary for SSRO. Al-Moraissi and Ellis [14] reported that both the bicortical screw and miniplate fixation methods show no significant difference in postsurgical skeletal stability. Thus, miniplate fixation was adopted in the present study.

Sorokolit and Nanda [15] indicated that a mean amount of surgical setback is approximately 5.1 mm and a mean amount of postsurgical anterior movement is approximately 0.51 mm, which suggests a 10% relapse. In another study, Chou et al. [16] reported that the immediate postsurgical Pog setback was 7 mm and 1 year later it was 5.5 mm; in other words, potency of relapse was 21%. Elsewhere, Costa et al. [17] conducted a literature review of SSRO surgeries used to correct mandibular prognathism and found that postsurgical relapses could be as high as 50%. Whether a significant relationship exists between the risk of relapse and the amount of setback remains controversial among researchers. In the present study, the relapse rate of Me was 42.5%, indicating that a statistically significant relationship existed between the risk of relapse and the amount of setback. Even when the reduced area of the PMS was significant, it still was not significant in relation to postoperative relapse.* Our finding was similar to the report of Lee et al. [12]; nonhigh FMA and high FMA of mandibular prognathism did not cause significant differences in the postoperation skeletal relapse.*

When the displacement amount is substantial, it is arguable whether the coronoid process needs to be removed. For example, Kruger Gustav [18] claimed that the coronoid process must be removed to treat severe mandibular prognathism because the temporal muscle is attached to the anterior border of the ramus and medially to the coronoid process. The coronoid process is a pivot that resists the amount of displacement required during surgery. However, a strong temporalis effect causes upward rotation of the coronoid process, thereby resulting in a postsurgical relapse. In the present study, the amount of setback was higher than that in previous studies; this could be the cause of the higher relapse rate in this study. Whether a two-jaw surgery (Le Fort I maxillary advancement and SSRO mandibular setback) should be conducted to reduce the amount of mandibular setback in a one-jaw surgery (SSRO mandibular setback) or the coronoid process should be removed to reduce the relapse rate merits further exploration.

Few studies have investigated frontal facial changes in patients after a mandibular prognathism setback surgery. Yoshioka et al. [19] found that, 1 year after SSRO surgery, the InterGo width of their studied patients increased by 0.45 mm. Meanwhile, Choi et al. [20] identified an increase of 2.1 mm in the InterGo width. In the present study, the InterRp and InterGo widths increased significantly by 1.8 and 2.2 mm, respectively. Additionally, the present study shows that InterRpS and InterGoS widths increased by 1.4 mm and 2.5 mm without significance, respectively. The increases in the InterRpS and InterGoS widths were 77.8% and 113.6% of InterRp and InterGo, respectively. Moreover, bilateral ramus angle increased by approximately 1° in the present study, representing minimal change in the ramus angles before and after the surgery.

Clinical observations have revealed that mandibular setback procedure slightly moves the cheek line forward. However, scarce information is available regarding changes in the cheek line. In the present study, the chin points anterior movements were approximately 0.21–0.36 mm, average cheek points (0.32 mm) representing a displacement percentage of 6% (average cheek points/Pog). This result can serve as a reference for overall changes in the profile. Notably, if the postsurgical relapse rate can be controlled, the cheek line curve can be markedly improved. Marşan et al. [6] observed that PogS/Pog ratios were 0.51. Mobarak et al. [5] reported that the PogS/Pog ratios were 1.04. Our result (PogS/Pog = 1.05) was similar to Mobarak et al. [5]. Yoshioka et al. [19] report that intergonial width and proximal segment angulations significantly increased to 2.1 mm and 1.8°. Similar to Choi et al. [20], our findings (InterGo widths and bilateral ramus angles) were increased significantly by 2.2 mm and 2°.

Postsurgical occlusion stability can minimize teeth movement and help prevent relapses. Therefore, surgeons and orthodontists must maintain smooth communication. When presurgical orthodontics cannot achieve stable postsurgical occlusion, the patient adjusts the occlusion to ease occlusal discomfort, thereby causing mandibular shift or anterior movement; these inappropriate movements can result in postsurgical relapses. It is thus critical to accurately predict soft and hard tissue movements, especially when proposing a treatment plan for orthognathic surgery. Accurate prediction can facilitate decision-making by orthodontists and surgeons and maintain favorable surgeon-patient communication. Furthermore, adequate postsurgical orthodontic therapy and maintenance of the mandibular position are common responsibilities of surgeons and orthodontists because the postsurgical bone healing and orthodontic treatment have interactive effects.

In summary, the amount of setback experienced following SSRO is significantly related to postoperative relapse. In particular, frontal transverse changes of the hard tissue were also significant.

## Figures and Tables

**Table 1:** Paired *t*-test for significance for the various cephalometric parameters (Me, Pog, and PogS) at the T21, T32, and T31 periods.

Parameters (mm)	Me			Pog			PogS		
	Mean	SD	*p* value	Mean	SD	*p* value	Mean	SD	*p* value
Horizontal change									
T21	−8.7	3.54	<0.001^*∗*^	−8.1	3.48	<0.001^*∗*^	−7.2	3.65	<0.001^*∗*^
T32	3.7	2.78	<0.001^*∗*^	2.8	2.09	<0.001^*∗*^	1.6	3.47	0.023^*∗*^
T31	−5.0	3.46	<0.001^*∗*^	−5.2	3.30	<0.001^*∗*^	−5.5	3.08	<0.001^*∗*^
Vertical change									
T21	0.4	2.05	0.454	−0.4	2.81	0.528	1.0	3.82	0.178
T32	−1.0	2.45	0.057	−1.1	2.08	0.016^*∗*^	−2.2	3.31	0.003^*∗*^
T31	−0.7	2.68	0.209	−1.4	2.82	0.017^*∗*^	−1.1	3.19	0.083

Me: menton; Pog: pognion; PogS: pognion at the soft tissue. ^*∗*^Significant  *p* < 0.05; T21: immediate surgical changes; T32: postoperative stability; T31: final surgical change.

**Table 2:** Cheek points, *FMA*, and pterygomasseteric sling's area at the final surgical change (T31).

Parameters	Mean	SD	*p* value
Cheek points (mm)			
C1	0.34	1.55	0.271
C2	0.36	1.55	0.246
C3	0.33	1.61	0.301
C4	0.35	1.55	0.259
C5	0.21	1.34	0.426
Average cheek points	0.32	1.47	0.277
*FMA (Total)*	*−0.22*	*2.37*	*0.637*
* High FMA *>* 29*°	*−0.57*	*2.15*	*0.356*
* Nonhigh FMA ≦ 29*°	*0.15*	*2.53*	*0.918*
Area of PMS (mm^2^)	−55.51	112.78	0.019^*∗*^

^*∗*^Significant  *p* < 0.05; T31: final surgical change; C1: +6 mm; C2: +3 mm; C3: 0 mm (origin point); C4: −3 mm; C5: −6 mm; *FMA: Frankfort-mandibular plane angle*; PMS: pterygomasseteric sling.

**Table 3:** Frontal landmarks at the final surgical change (T31).

Parameters (mm)	Right side			Left side		
	Mean	SD	*p* value	Mean	SD	*p* value
Horizontal change						
Rp	0.81	2.07	0.055	1.02	2.14	0.023^*∗*^
RpS	0.74	4.15	0.371	0.67	3.42	0.329
Go	1.15	3.46	0.102	1.06	2.63	0.051
GoS	1.59	4.32	0.071	0.91	3.63	0.214
Vertical change						
Rp	0.61	6.09	0.613	0.89	5.81	0.443
Go	−1.59	5.81	0.174	−2.43	5.61	0.037^*∗*^

^*∗*^Significant, *p* < 0.05; Rp: ramus posterior; RpS: Rp at the soft tissue; Go: gonion; GoS: Go at the soft tissue.

**Table 4:** Transverse measurements at final surgical change (T31).

Parameters (mm)	T31		
	Mean	SD	*p* value
Frontal width (mm)			
InterRp	1.8	3.36	0.010^*∗*^
InterRpS	1.4	6.88	0.307
InterGo	2.2	4.06	0.010^*∗*^
InterGoS	2.5	6.74	0.070
Frontal angle (degree)			
Right side			
Ramus angle	1.0	4.12	0.219
RamusS angle	1.9	5.34	0.083
Left side			
Ramus angle	1.0	2.32	0.034^*∗*^
RamusS angle	1.1	4.17	0.193

^*∗*^Significant, *p* < 0.05; Rp: ramus posterior; RpS: Rp at the soft tissue; Go: gonion; GoS: Go at the soft tissue; RamusS: ramus at the soft tissue; PMS: pterygomasseteric sling.

**Table 5:** Pearson correlation testing for postoperative stability (T32).

Parameters (mm)	Me			
	Horizontal (T32)		Vertical (T32)	
	*r*	*p* value	*r*	*p* value
Horizontal change				
Me (T21)	−0.423	0.028^*∗*^	0.327	0.096
Pog (T21)	−0.211	0.292	−0.063	0.755
Right Rp (T31)	0.128	0.525	0.081	0.688
Right Go (T31)	0.095	0.638	−0.059	0.770
Left Rp (T31)	0.190	0.343	−0.324	0.099
Left Go (T31)	0.091	0.653	−0.515	0.006^*∗*^
Vertical change				
Me (T21)	0.173	0.387	−0.300	0.128
Pog (T21)	0.046	0.820	0.011	0.958
Right Rp (T31)	0.210	0.292	−0.420	0.029^*∗*^
Right Go (T31)	0.107	0.594	−0.528	0.005^*∗*^
Left Rp (T31)	0.190	0.342	−0.384	0.048^*∗*^
Left Go (T31)	0.091	0.653	−0.362	0.064
InterRp (T31)	0.199	0.319	−0.157	0.435
InterGo (T31)	0.139	0.488	−0.383	0.049^*∗*^
Right ramus angle (T31)	0.081	0.689	−0.015	0.940
Left ramus angle (T31)	0.007	0.972	−0.500	0.008^*∗*^
*FMA (T31)*	*−0.075*	*0.710*	*−0.198*	*0.322*
Area of PMS (mm^2^) (T31)	−0.073	0.717	0.114	0.571

^*∗*^Significant  *p* < 0.05; *r*: correlation coefficient; T21: immediate postoperative changes; T32: postoperative stability; T31: final surgical change; *FMA: Frankfort-mandibular plane angle*; PMS: pterygomasseteric sling.

## References

[1] Kang H.-K., Ryu Y.-K. (1992). A study on the prevalence of malocclusion of Yonsei University students in 1991. *Korean Journal of Orthodontics*.

[2] Tang E. L. (1994). The prevalence of malocclusion amongst Hong Kong male dental students. *British Journal of Orthodontics*.

[3] Thilander B., Myberg N. (1973). The prevalence of malocclusion in Swedish school children. *Scandinavian Journal of Dental Research*.

[4] Jacobson A., Evans W. G., Preston C. B., Sadowsky P. L. (1974). Mandibular prognathism. *American Journal of Orthodontics and Dentofacial Orthopedics*.

[5] Mobarak K. A., Krogstad O., Espeland L., Lyberg T. (2001). Factors influencing the predictability of soft tissue profile changes following mandibular setback surgery. *The Angle Orthodontist*.

[6] Marşan G., Öztaş E., Kuvat S. V., Cura N., Emekli U. (2009). Changes in soft tissue profile after mandibular setback surgery in Class III subjects. *International Journal of Oral and Maxillofacial Surgery*.

[7] Ingervall B., Thüer U., Vuillemin T. (1995). Stability and effect on the soft tissue profile of mandibular setback with sagittal split osteotomy and rigid internal fixation. *International Journal of Adult Orthodontics & Orthognathic Surgery*.

[8] Joss C. U., Vassalli I. M., Thüer U. W. (2008). Stability of soft tissue profile after mandibular setback in sagittal split osteotomies: a longitudinal and long-term follow-up study. *Journal of Oral and Maxillofacial Surgery*.

[9] Chen C.-M., Chen M. Y.-C., Cheng J.-H., Chen K.-J., Tseng Y.-C. (2017). Facial profile and frontal changes after bimaxillary surgery in patients with mandibular prognathism. *Journal of the Formosan Medical Association*.

[10] Burstone C. J., James R. B., Legan H., Murphy G. A., Norton L. A. (1978). Cephalometrics for orthognathic surgery. *Journal of Oral Surgery*.

[11] Lee D.-H., Yu H.-S. (2012). Masseter muscle changes following orthognathic surgery: A long-term three-dimensional computed tomography follow-up. *The Angle Orthodontist*.

[12] Lee Y.-S., Kim Y.-K., Yun P.-Y., Larson B. E., Lee N.-K. (2016). Comparison of the stability after mandibular setback with minimal orthodontics of class iii patients with different facial types. *Journal of Oral and Maxillofacial Surgery*.

[13] Komori E., Aigase K., Sugisaki M., Tanabe H. (1987). Skeletal fixation versus skeletal relapse. *American Journal of Orthodontics and Dentofacial Orthopedics*.

[14] Al-Moraissi E. A. M., Ellis E. (2016). Stability of bicortical screw versus plate fixation after mandibular setback with the bilateral sagittal split osteotomy: A systematic review and meta-analysis. *International Journal of Oral and Maxillofacial Surgery*.

[15] Sorokolit C. A., Nanda R. S. (1990). Assessment of the stability of mandibular setback procedures with rigid fixation. *Journal of Oral and Maxillofacial Surgery*.

[16] Chou J. I.-C., Fong H.-J., Kuang S.-H. (2005). A retrospective analysis of the stability and relapse of soft and hard tissue change after bilateral sagittal split osteotomy for mandibular setback of 64 Taiwanese patients. *Journal of Oral and Maxillofacial Surgery*.

[17] Costa F., Robiony M., Politi M. (2001). Stability of sagittal split ramus osteotomy used to correct Class III malocclusion: review of the literature. *International Journal of Adult Orthodontics & Orthognathic Surgery*.

[18] Kruger Gustav O. (1984). Developmental deformities of the jaws. *Textbook of Oral and Maxillofacial Surgery*.

[19] Yoshioka I., Khanal A., Tominaga K., Horie A., Furuta N., Fukuda J. (2008). Vertical ramus versus sagittal split osteotomies: comparison of stability after mandibular setback. *Journal of Oral and Maxillofacial Surgery*.

[20] Choi H.-S., Rebellato J., Yoon H.-J., Lund B. A. (2005). Effect of mandibular setback via bilateral sagittal split ramus osteotomy on transverse displacement of the proximal segment. *Journal of Oral and Maxillofacial Surgery*.

